# Salt-Starvation in Epilepsy

**Published:** 1901-08-10

**Authors:** 


### SALT-STARVATION IN EPILEPSY.

No one can watch cases of epilepsy through long periods without becoming impressed with the importance of their diet. Again and again it happens that a long interval of freedom from attacks is broken as the apparent result of some indiscretion or some excess. In fact it may truthfully be said that the modes by which we can exercise an influence over this disease are mainly four—occupation, diet, attention to the bowels, and drugs—and that although, for the production of an immediate effect, drugs are perhaps of the greatest efficacy, the other means are, in the long run, of the greatest utility, and none more so than diet. As a combination of a dietetic and medicinal treatment, we would draw attention to the method of "salt starvation" advocated by Richet, combined with the administration of small quantities of bromides to take the place of the chloride of sodium so eliminated from the diet. A paper recently appeared in the *Berliner Klinische Wochenschrift* describing the effect of such a diet upon 28 patients who were subject to idiopathic epilepsy (*petit mal* and *haut mal*). All chlorides were as far as possible eliminated from the diet, and then a small quantity of bromides was added. The following was the dietary which after various trials was found to meet the conditions: From one to one and a half litres of milk, from 40 to 50 grammes of butter, three eggs, and from 300 to 400 grammes of bread and fruit, the bread being specially prepared without common salt, but with the addition of 3 grammes of bromide of sodium per loaf. It was calculated that living on this diet only about 2 grammes of sodium chloride per diem would be taken, while the patient would all the time be taking, unconsciously, about 3 grammes of sodium bromide. The results obtained are stated to have been highly satisfactory in all the cases, a reduction in the frequency and violence of the attacks together with an improvement in the mental condition being noted. The general health improved and bromism was not produced. Certainly such a line of treatment is worthy of trial in cases in which the diet can be regulated, as, for example, in institutions where the special bread required can easily be supplied. We should, however, have more confidence in the results of "salt starvation" if each half of this experiment had been tried separately. No dietetic experiment which involves the administration of 45 grains of bromide per day can be regarded as very conclusive, knowing as we do how effective, for a time, even a mere change from one sort of bromide to another may be in this most fickle malady.

